# An international, multicentre, interventional, randomised, assessor-blinded trial to MAXimise the METHotrexate therapy potential in patients with active rheumatoid arthritis (MethMax trial): study protocol for a randomised controlled trial

**DOI:** 10.1186/s13063-026-09519-4

**Published:** 2026-03-23

**Authors:** Karolina Anderle, Daniela Sieghart, Martina Durechova, François Bonnay, Andreas Kerschbaumer, Katerina Chatzidionysiou, Rachel Knevel, Costantino Pitzalis, Siri Lillegraven, Espen A. Haavardsholm, Eirik Klami Kristianslund, Catalin Codreanu, Claudiu Popescu, Elisa Gremese, Sabina de Geest, Agnes Kocher, Souzi Makri, Daniel Aletaha, Helga Lechner-Radner, the SQUEEZE Consortium

**Affiliations:** 1 Division of Rheumatology, Department of Internal Medicine III, Medical University of Vienna, Guertel 18-20, Vienna, A-1090 Austria; 2 Department of Medicine, Solna, Division of Rheumatology, Karolinska University Hospital at Karolinska Institutet, Stockholm, Sweden; 3 Department of Rheumatology, Leiden University Medical Center, Leiden, Netherlands; 4 Centre for Experimental Medicine and Rheumatology, William Harvey Research Institute, Barts and The London School of Medicine and Dentistry, Queen Mary University of London & NIHR BRC Barts Health NHS Trust, London, UK; 5 Department of Rheumatology, Diakonhjemmet Hospital, Oslo, Norway; 6 Faculty of Medicine, University of Oslo, Oslo, Norway; 7 Center for Rheumatic Diseases, Carol Davila University of Medicine and Pharmacy, Bucharest, Romania; 8 Rheumatology and Clinical Immunology, IRCCS Humanitas Research Hospital, Rozzano, Milan Italy; 9 Department of Biomedical Sciences, Humanitas University, Pieve Emanuele, Milan, Italy; 10 Nursing Science, Department Public Health, Faculty of Medicine, University of Basel, Basel, BS Switzerland; 11 Patient Research Partner, EULAR, Nicosia, Cyprus

**Keywords:** Methotrexate, Rheumatoid arthritis, Patient-reported outcomes, Clinical disease activity, Explorative biomarkers, Adherence, Digital health

## Abstract

**Background:**

Methotrexate (MTX) is recommended as first-line therapy in patients with rheumatoid arthritis (RA), proven to be effective, safe and inexpensive. However, a significant proportion of patients does not achieve disease remission with MTX monotherapy. Main reasons include insufficient dose up-titration to the maximal recommended oral dose or the delayed switch to a subcutaneous administration route. We hypothesise, that by dose and route optimisation, a higher proportion of patients can achieve remission. Further, exploratory biomarkers will give new insights on individual MTX metabolism and drug adherence.

**Methods:**

The MethMax trial is a prospective, randomised, assessor-blinded, parallel-group, superiority, low-intervention trial, including 182 patients across 7 European countries. Patients with active RA, naïve to biologic (except tumour necrosis factor alpha inhibitors; TNFi) or targeted synthetic antirheumatic drugs, who have been on a stable oral MTX therapy for the past 3 months are randomised in a 1:1 ratio to 25 mg MTX weekly, either administered orally or subcutaneously. Additionally, both arms receive a short-term glucocorticoid regimen with a four-week tapering and withdrawal protocol. The primary endpoint is the difference in proportion of patients achieving remission defined as the Clinical Disease Activity Index (CDAI) ≤ 2.8 at week 24, comparing the dose/route optimisation and oral dose optimisation. The active study duration for each patient is 24 weeks. Study visits take place at baseline, weeks 4, 12, 16 and 24. Clinical efficacy and safety parameters are obtained at each visit. Patient-reported outcomes, exploratory biomarkers as well as medication adherence are assessed. Written consent is obtained for all participants. The study has received regulatory approval via the Clinical Trials Information System and Medicines and Healthcare products Regulatory Agency and has included the first patient in August 2024.

**Discussion:**

The anticipated results will provide insights whether the subcutaneous administration of 25 mg MTX is advantageous in achieving CDAI remission when compared to the oral intake after 24 weeks and inform the community regarding the utility of established and newly developed biomarkers, as well as the potential impact of inadequate drug adherence.

The MethMax study is aimed to optimise individual therapy in RA and provide more precise pharmacological MTX management recommendations.

## Structured summary {1b}

**Table:** 

Item	Description
Primary Registry and Trial Identifying Number {4}	ClinicalTrials.gov identifier NCT06649136, registered on October 17, 2024.
Secondary Identifying Numbers	Clinical Trials Information System (CTIS), identifier EU-CT Number 2023–507714-27–00
Source(s) of Monetary or Material Support	• The European Union (Horizon Europe) under grant agreement no. 101095052 (SQUEEZE) • UK Research and Innovation (UKRI) under the UK government’s Horizon Europe funding guarantee grant no. 10055567 • Swiss State Secretariat for Education, Research and Innovation (SERI) • Additional funding was provided to Leiden University Medical Center by the SPIDeRR HORIZON EU Grant No 101080711.
Primary Sponsor and contact information {3b}	Medical University of Vienna, Department of Internal Medicine III, Division of Rheumatology Waehringer Guertel 18–20, A-1090 Vienna, Austria Phone: + 43 1 40400–43010, Email: helga.lechner-radner@meduniwien.ac.at
Role of sponsor and funder {3c}	The sponsor is an academic institution and is responsible for all aspects of the study, including its design; collection, management, analysis, and interpretation of data; writing of the report; and the decision to submit the report for publication. The funder has no role independent of the sponsor, and the sponsor retains full authority over all trial-related activities. The coordinating centre and steering committee are based at the Medical University of Vienna and are responsible for overall trial management, protocol oversight, and decision-making. No separate endpoint adjudication committee is planned. Data management is conducted by the sponsor institution.
Contact for Public Queries	https://innere-med-3.meduniwien.ac.at/en/unsere-abteilungen/rheumatologie/research/clinical-trial-unit/ methmax@meduniwien.ac.at
Contact for Scientific Queries	Assoc. Prof. Priv. Doz. Dr. Helga Lechner-Radner Medical University of Vienna, Department of Internal Medicine III, Division of Rheumatology Waehringer Guertel 18–20, A-1090 Vienna, Austria Phone: + 43 1 40,400–43010, Email: helga.lechner-radner@meduniwien.ac.at, methmax@meduniwien.ac.at
Public Title	MAXimising the METHotrexate therapy potential in patients with active rheumatoid arthritis (MethMax trial)
Scientific title	An international, multicentre, interventional, randomised, assessor-blinded trial to MAXimise the METHotrexate therapy potential in patients with active rheumatoid arthritis (MethMax trial)
Countries of Recruitment	Austria, Italy, Netherlands, Norway, Romania, Sweden, United Kingdom
Health Condition(s) or Problem(s) Studied	Rheumatoid Arthritis
Intervention(s)	Adapting the ongoing methotrexate therapy to either 25 mg subcutaneously once weekly or 25 mg orally once weekly
Key Inclusion Criteria	Adult patients diagnosed with rheumatoid arthritis who have active disease, at least one swollen joint and have been receiving stable MTX therapy at a dose of 10–25 mg for a minimum of 3 months.
Key Exclusion Criteria	1. Inflammatory rheumatic diseases other than RA 2. Ongoing or previous therapy with any targeted synthetic DMARDs (tsDMARD) or biological Disease-Modifying Anti-Rheumatic Drug (bDMARD) with the exception of any TNFα inhibitor in a stable dose and interval for at least 4 months; We allow ongoing or previous therapy with a conventional synthetic DMARD (csDMARD) with a stable dose in the past 4 months. 3. Use of GC unless on stable oral dose ≤ 10 mg for at least 4 weeks prior to study inclusion 4. Patients using NSAIDs, unless taken at a stable dose for ≥ 2 weeks prior to study inclusion 5. Intraarticular GC treatment in the last 8 weeks 6. Patients with significant and clinically relevant MTX-drug toxicity as judged by the investigator Further exclusion criteria define organ function thresholds, infections, vaccinations, participation in studies and other (full list is provided in section 14a).
Study Type	Prospective, international, multicentre, randomised, assessor-blinded, parallel-group, superiority, low intervention study Phase IV
Date of First Enrollment (planned)	12-Aug-2024
Sample Size	182
Primary outcome(s)	The primary endpoint is the achievement of remission defined as the CDAI ≤ 2.8 assessed 24 weeks after randomisation comparing patients with dose/route optimisation (≥ 10 mg MTX oral weekly switched to 25 mg MTX subcutaneously weekly) and oral dose optimisation (≥ 10 mg MTX oral weekly switched to 25 mg MTX oral weekly).
Key Secondary outcome(s)	The proportion of patients achieving CDAI low disease activity, American College of Rheumatology (ACR) scores ACR20, ACR50, ACR70, patient reported outcomes and inflammatory markers across different timepoints. Exploratory endpoints include assessment of methotrexate-polyglutamates (MTX-PGs), sweat metabolites, treatment adherence, safety parameters and cumulative glucocorticoid dose.
Ethics Review	Each country specific ethics review was processed via CTIS, identifier EU-CT Number 2023–507714-27–00. In detail, the Ethics Committee of the Medical University of Vienna was responsible for review of the main trial sponsor site, Medical University of Vienna.
Individual Trial Participant Data sharing statement	Deidentified individual trial participant data can be provided upon reasonable request.

## Protocol version {2}

Version 2.0, 16th May 2025.

## Introduction

### Background and rationale {9a, b}

Methotrexate (MTX) is recommended as the disease-modifying antirheumatic drug (DMARD) of choice in patients newly diagnosed with rheumatoid arthritis (RA) [1]. It is considered an anchor drug for RA given its well-established and balanced efficacy, safety and tolerability profile, but also its relative affordability. MTX is a folic acid analogue whose immunomodulatory effects are mediated through multiple mechanisms, including inhibition of folate-dependent enzymes such as dihydrofolate reductase and ATIC (AICAR transformylase), leading to intracellular AICAR accumulation and increased extracellular adenosine release [2, 3]. Furthermore, concomitant folate substitution provides increased safety and reduces side effects [4]. Despite all its advantages, achieving long-term remission with MTX monotherapy is only limited to a minority of RA patients [5]. The main known limitations in clinical long-term use of this drug are (a) the insufficient titration to the maximum recommended per os (p.o.) dose of 25 mg weekly [6] and (b) the delayed switch to subcutaneous (s.c.) route or an addition of a biologic or targeted synthetic therapy when the oral route is deemed ineffective. Moreover, adherence to MTX might be influenced by relatively common side effects associated with oral administration such as nausea, abdominal discomfort or headache.

Existing studies provided a reasonable basis for s.c. application of MTX (SCMTX) being superior to oral administration (POMTX), leading to improved efficacy [7–9]. SCMTX application showed higher efficacy due to improved bioavailability and reduced side effects (gastrointestinal adverse events (AEs) reduced by 30%) in previous trials [10, 11]. According to the European Alliance of Associations for Rheumatology (EULAR) guidelines for the use of MTX, both MTX dose and route should be optimised to achieve maximum effectiveness, provided no safety concerns arise. This is particularly relevant for patients who have already shown an initial positive response to MTX: such early treatment response may serve as a marker to identify patients who may benefit most from dose or route optimisation [12].

MTX monotherapy is often escalated to a TNFi if MTX alone fails to achieve adequate clinical response or disease control. TNFi, when used in combination with MTX, have demonstrated superior efficacy compared to TNFi monotherapy in the treatment of RA [13, 14]. MTX not only enhances the clinical effectiveness of TNFi but also helps reduce the formation of anti-drug antibodies (ADAs), thereby improving drug persistence and treatment durability [15–17]. The concomitant use of MTX is particularly important in maintaining therapeutic drug levels, minimising immunogenicity, and delaying secondary loss of response. This combination strategy has become a cornerstone in optimising biologic therapy outcomes.

The metabolic pathway of MTX is complex. In hepatocytes, aldehyde oxidases convert MTX to 7-hydroxymethotrexate (7-OH-MTX), a metabolite that has been proposed to influence MTX efficacy [18]. When transported intracellularly, MTX is converted into active MTX polyglutamates (MTX-PGs) by folylpolyglutamate synthetases, which add glutamate residues to MTX in multiple steps [19]. Importantly, while laboratory measurements of active metabolites (MTX-PGs 1–7) are methodologically challenging, their association with disease activity and patient characteristics is extremely valuable to better understand MTX treatment outcomes. Moreover, MTX-PGs could also be used as adherence measurements for patients with ongoing MTX treatment [20, 21].

Therapeutic drug monitoring (TDM) is applied in multiple clinical fields, e.g. in neuropsychiatric [22] and antimicrobial [23] therapy, as well as in biologic therapy in inflammatory bowel disease [24]. Importantly, however, TDM is insufficiently documented in rheumatic diseases, and the use of proactive TDM is currently not recommended for biological DMARDs (bDMARDs), other than infliximab [17, 25, 26]. This has prompted paramount research in TDM for biologics [26] and synthetic DMARDs [27] in rheumatic diseases.

Finally, we should also consider intra-individual variation of immunosuppression levels, which are influenced by multiple factors, making any quantification difficult. This aspect was recently investigated via measuring peripheral blood levels of torque teno virus (TTV), a virus found in the majority of the healthy adult population, which is associated with rejection and infection in solid organ transplant recipients [28]. Recent data showed a possible use of TTV measurement in patients with RA on bDMARDs to better determine their immunosuppression level [29]. This has prompted us to investigate TTV in this trial, for the first time in patients receiving MTX.

Here, we describe the study protocol of the MethMax trial—which is aimed to evaluate the clinical and biomarker response after optimising the MTX treatment regimen. We hypothesise that in RA patients with active disease despite ongoing MTX treatment, SCMTX is superior to POMTX at weekly dosing of 25 mg after 24 weeks. Furthermore, we want to explore the utility of novel biomarkers (metabolites in blood and sweat) in these patients.

### Objectives {10}

The primary objective is to assess the proportion of patients in CDAI remission (≤ 2.8) at week 24. Among the secondary objectives, we assess the proportion of patients achieving CDAI low disease activity, ACR20, ACR50, ACR70, patient-reported outcomes and inflammatory markers across different timepoints. Exploratory objectives include assessment of MTX-PGs, sweat metabolites, treatment adherence, safety parameters and cumulative GC dose.

## Methods: patient and public involvement, and trial design

### Patient and public involvement {11}

The study aims to enrol 182 patients across 7 European countries, at 7 study sites. Eligible patients need to be classified as having RA based on the ACR/EULAR 2010 classification criteria [30] with ongoing stable oral MTX therapy at a dosage of at least 10 mg/week to a maximum of 25 mg/week for at least 12 weeks. For inclusion, patients need to have active disease as assessed by the CDAI of > 2.8 AND at least one or more clinically swollen joints on a 28-joint count. History of any csDMARD and TNFi and their concomitant use in a stable dosing regimen is allowed. Concomitant treatment with glucocorticoids and non-steroidal anti-inflammatory drugs (NSAIDs) in a stable dosing regimen is allowed as described below.

### Trial design {12}

The MethMax trial is a prospective, international, multicentre, randomised, assessor-blinded, parallel-group, superiority, low intervention trial. The study follows the principles of the International Council for Harmonisation of Technical Requirements for Registration of Pharmaceuticals for Human Use—Good Clinical Practice (ICH GCP), the Declaration of Helsinki and complies with respective country-specific regulations. Before study inclusion, written informed consent is obtained from each participant. The protocol of the MethMax study fulfils the requirements of the SPIRIT statement (Standard Protocol Items: Recommendations for Interventional Trials).

## Methods: participants, interventions and outcomes

### Trial setting {13}

The trial is one of the three clinical trials within the Squeeze project, funded by the Horizon Europe Programme. It has gained regulatory approval in the European Union through the Clinical Trials Information System (CTIS) with the identifier EU-CT Number 2023–507714-27–00 for the following six centres: Medical University of Vienna, Vienna, Austria (29th April 2024); Leiden University Medical Centre, Leiden, Netherlands (22nd April 2024); Diakonhjemmet Hospital, Oslo, Norway (22nd April 2024); Karolinska University Hospital, Stockholm, Sweden (25th April 2024); Carol Davila University of Medicine and Pharmacy, Bucharest, Romania (3rd February 2025) and Humanitas Research Hospital, Milan, Italy (9th May 2025). The regulatory approval for Queen Mary University of London (QMUL), London, UK, was granted on 15th April 2025 by the Medicines and Healthcare products Regulatory Agency (MHRA) and the Research Ethics Committee. The trial was registered on ClinicalTrials.gov, with the identifier NCT06649136 at the time of patient enrolment start. The original protocol, version 1.1, was amended to version 2.0 in May 2025 via CTIS and was granted approval in August 2025. The changes to the protocol are summarised in the “Protocol amendments” section.

## Eligibility criteria for participants {14a}

The inclusion criteria are as follows:

1. Men and women, ≥ 18 years of age, capable of understanding and signing an informed consent (including sufficient literacy and proficiency in the local language) and following the study procedures.
2. Patients with RA according to the 2010 ACR/EULAR classification criteria.
3. Ongoing conventional therapy with oral MTX (between ≥ 10 mg and 25 mg weekly) for ≥ 3 months with stable dosing, and clinical and laboratory tolerance of this treatment for at least 12 weeks.
4. CDAI > 2.8 + at least 1 clinically swollen joint (on 28-joint count).
5. Willingness to increase MTX dosing and change the route of administration according to study procedures.

The exclusion criteria are as follows:

1. Inflammatory rheumatic diseases other than RA.
2. Ongoing or previous therapy with any targeted synthetic DMARDs (tsDMARD) or biological Disease-Modifying Anti-Rheumatic Drug (bDMARD) with the exception of any TNFα inhibitor in a stable dose and interval for at least 4 months. We allow ongoing or previous therapy with a conventional synthetic DMARD (csDMARD) with a stable dose in the past 4 months.
3. Use of glucocorticoids (GC) unless on stable oral dose ≤ 10 mg for at least 4 weeks prior to study inclusion.
4. Patients using NSAIDs, unless taken at a stable dose for ≥ 2 weeks prior to study inclusion.
5. Intraarticular GC treatment in the last 8 weeks.
6. Patients with significant and clinically relevant MTX-drug toxicity as judged by the investigator.
7. Elevated liver enzymes (aspartate transaminase (AST) and/or alanine transaminase (ALT)), and/or alkaline phosphatase (AP), and/or gamma-glutamyl transferase (GGT) above two times the upper limit normal (ULN).
8. Reduced kidney function (glomerular filtration rate (GFR) < 60 mL/min/1.73 m^2^).
9. Haematologic abnormalities (grade 2 or 3: anaemia, leukopenia, thrombocytopenia).
10. Stomatitis under treatment with MTX.
11. Known history of recurrent/serious infections in the previous 2 months (such as, but not limited to, hepatitis, pneumonia, or pyelonephritis).
12. A positive HBs antigen and/or hepatitis C virus (HCV) test at screening visit.
13. Ongoing or recurring opportunistic infections (e.g. herpes zoster, cytomegalovirus, pneumocystis, aspergillosis, histoplasmosis, or mycobacteria) as judged by the investigator.
14. Women of childbearing potential without the use of adequate birth control measures (e.g. abstinence, oral contraceptives, intrauterine device, barrier method with spermicide, implantable or injectable contraceptives or surgical sterilisation) and willing to continue this precaution for the duration of the study until 6 months after receiving the last medication.
15. Current signs or symptoms of severe, progressive or uncontrolled renal, hepatic, haematologic, gastrointestinal, endocrine, pulmonary, cardiac, neurologic, or cerebral disease, as judged by the investigator.
16. Being unable or unwilling to undergo multiple venipunctures because of poor tolerability or lack of sufficient venous access.
17. Being unwilling or unable to perform s.c. injections.
18. Presence of a transplanted solid organ (except for a corneal transplant > 3 months prior to screening).
19. Women who are pregnant, nursing or planning pregnancy during the study and 6 months after the individual study completion.
20. History of alcohol or substance abuse within the preceding 6 months.
21. Any medical or psychological condition that, judged by the investigator, would interfere with the safe completion of the trial.
22. Immunisation with a live/attenuated vaccine within 12 weeks prior to baseline or potential need to receive a live vaccine during the course of the study.
23. Active participation in any other interventional study.

### Who will take informed consent? {32a}

Written informed consent will be obtained by a trained study investigator from all participants prior to any study-specific procedures, after provision of comprehensive verbal and written information about the study and adequate time for discussion.

### Additional consent provisions for collection and use of participant data and biological specimens {32b}

Participants will be asked to provide additional, voluntary consent for the collection and use of health-related data via a mobile application RheumaBuddy. This consent is obtained separately from the main study informed consent and refusal does not affect participation in the study or access to study procedures. Additional voluntary consent is sought for ancillary studies involving optional collection of Peripheral Blood Mononuclear Cells (PBMCs) and storage in local biobanks at participating centres, using centre-specific biobank consent forms.

## Intervention and comparator

### Intervention and comparator description {15a}

Patients with RA receiving MTX who are not on target at baseline visit (CDAI ≤ 2.8) are randomised either to a locally available generic SCMTX injection, 25 mg/week, or locally available generic POMTX tablets, 25 mg/week in total. All patients receive an initial GC tapering regimen for 4 weeks, starting with 20 mg prednisone equivalent daily. After 4 weeks, the GC are tapered to 0 in patients with no GC intake at study inclusion, or to the baseline dose for patients who were enrolled in the study with a concomitant GC therapy of up to 10 mg daily.

### Criteria for discontinuing or modifying allocated intervention/comparator {15b}

Criteria for discontinuation or modification of the allocated intervention or comparator include safety concerns, serious or persistent adverse events, pregnancy, protocol deviations affecting participant safety, or investigator judgement. Participants may withdraw consent at any time without consequences for further treatment or study participation.

In case of a worsening of disease activity (CDAI increase > 4.5 compared to baseline) at each visit from week 4 onwards or inadequate improvement of the sum of swollen and tender joints (< 20% change compared to baseline) from week 12 onwards, rescue treatment applies according to standard of care (such as switch or add on of conventional synthetic, biologic or targeted synthetic DMARD (cs/b/tsDMARD)). This results in the participant’s early termination from the study. Non-responder imputation will apply to all patients with a premature end of study.

In case of an AE, a diagnostic or therapeutic procedure, an abnormal assessment (e.g. laboratory abnormalities) the MTX dose can be temporarily interrupted or reduced to ≥ 10 mg/week and re-escalated to 25 mg after resolution of the problem. If drug interruption or intermittent reduction is within a timeframe of ≤ 3 weeks, and re-institution of full dose of the study drug is safe according to the decision of the investigator, no permanent study discontinuation is required.

### Strategies to improve adherence to intervention/comparator {15c}

Adherence to the allocated intervention or comparator is promoted through a combination of participant education, regular follow-up, and monitoring procedures. Patient adherence to the study intervention is assessed using three different methods, described below in the “Intervention and comparator”section; the RheumaBuddy mobile application, a paper-based questionnaire (BAASIS-MTX), and the measurement of biomarkers.

### Concomitant care permitted or prohibited during the trial {15d}

All participants will receive an open-label short-term GC pulse at baseline according to standard of care for active RA on MTX. The prednisone-equivalent dose and a standardised tapering scheme will be prescribed by the investigator and initiated on day 1 after baseline. GC intake during the first 4 weeks will be recorded in a patient diary. GC use beyond the initial taper will be documented at each visit.

Folate supplementation will be prescribed as standard of care for MTX therapy according to a predefined scheme and recorded as concomitant medication.

Permitted concomitant medications.

- TNFα inhibitors (stable ≥ 4 months)
- csDMARDs (stable ≥ 4 months)
- NSAIDs and analgesics at investigator’s discretion

Prohibited concomitant medications.

Initiation or dose escalation of immunomodulatory therapies. Introduction of additional cs/b/tsDMARDs, or other immunosuppressants will lead to discontinuation of the investigational product.

Participants will be instructed not to initiate any new medication without prior consultation with the investigator.

### Ancillary and post-trial care {34}

A voluntary follow-up visit occurs 4 weeks after premature discontinuation of the study drug, during which clinical disease activity and relevant laboratory safety and inflammatory parameters are assessed. After completion of the trial, participants return to routine standard-of-care follow-up.

## Outcomes {16}

The detailed description of primary, secondary and explorative endpoints is provided in Table 1.

**Table 1:** Endpoints and definitions

Endpoint	Definition
**Primary endpoint**	
CDAI remission at week 24	Achievement of remission defined as the Clinical Disease Activity Index (CDAI) ≤ 2.8 assessed 24 weeks after randomisation comparing patients with dose/route optimisation and oral dose optimisation
**Secondary endpoints**	
CDAI activity and ACR response at different timepoints	To assess the proportion of patients: -In CDAI low disease activity (≤ 10) at weeks 24 and 12 -In CDAI remission (≤ 2.8) at week 12 -Achieving an ACR20, ACR50, ACR70 response at week 24 and 12 respectively
Patient reported outcomes	Difference in change (absolute and relative) of patient reported outcomes, including pain, patient global assessment, FACIT-F, SF36v1 and HAQ-DI between the treatment groups between baseline and week 24 and 12 respectively
Clinical and laboratory disease markers	Difference in change (absolute and relative) of swollen joint count, tender joint count, and C-reactive protein (CRP)/erythrocyte sedimentation rate (ESR) at week 24 between the treatment groups between baseline and week 24 and 12 respectively
**Exploratory endpoints**	
TDM in blood	Association of MTX-PGs levels and CDAI response at week 12 and week 24
	Association of MTX dosage, MTX-PGs levels and TTV titre as a potential marker to guide levels of immunosuppressive therapy at week 12 and week 24
Sweat analysis	Association between treatment-associated biomarker profiles and longitudinal clinical outcomes
GC dose	Difference in cumulative GC dose between treatment arms
Treatment adherence	Association of CDAI response and treatment adherence as measured by paper-based questionnaires, MTX metabolites and electronic adherence monitoring (in patients using the RheumaBuddy mobile app)
Safety parameters	Differences in safety profile according to number of AEs and organ systems (haematologic, hepatic, gastrointestinal, infections)
Disease trajectory	Trajectories of disease activity in the two groups over all visits, and its relation to predictors

### Exploratory laboratory assessments

MTX-PGs, MTX in plasma and the 7-OH-MTX are assessed at baseline, weeks 4, 12 and 24. One EDTA blood sample tube is taken, inverted 5 times and put immediately on crushed ice for exactly 30 min before centrifugation for 15 min at 4 °C and 2.000 × g. From this sample, the plasma and RBC pellet aliquots are stored at − 80 °C and regularly shipped on dried ice to the Medical University of Vienna for analysis. The MTX-PG 2–7 are analysed from the RBC pellet aliquots with a liquid chromatography-electrospray ionisation-tandem mass spectrometry-based assay using stable-isotope-labelled internal standards. The concentrations of MTX-PGs are reported in nmol/L packed erythrocytes.

One plasma aliquot collected at baseline, weeks 4, 12 and 24 is used for extraction of the viral DNA for the TTV analysis, using polymerase chain reaction at the Leiden University Medical Centre.

The sweat samples are obtained non-invasively from finger sweat using filter paper. The patients are instructed on sweat sampling method at the baseline visit, perform the first sampling under the supervision of study personnel, and the subsequent samples at home according to a diary. The samples are analysed at the Department of Analytical Chemistry at the University of Vienna.

### Adherence assessments

To assess treatment adherence, the RheumaBuddy application, a paper-based questionnaire (BAASIS-MTX), and the measurement of biomarkers are used.

Patients are introduced to RheumaBuddy, an existing Health App (www.rheumabuddy.com) and may choose to use it in accordance with its terms and conditions. Consenting patients are registered during baseline visit by using a study and centre specific code. In the MethMax-adapted version of the application, patients will receive weekly reminder to fill in information on painful joints on a homunculus, disease related symptoms on a numeric rating scale, and last MTX intake. Optionally, patients can report symptoms more frequently, in a need-adapted manner.

At each study visit, a paper-based questionnaire is used to assess adherence. For this purpose, a validated BAASIS© questionnaire for transplanted patients on immunosuppressive therapy (https://baasis.nursing.unibas.ch/) was adapted for the MethMax trial = BAASIS-MTX.

### Harms {17}

MTX is associated with a well-characterised adverse event profile, including gastrointestinal symptoms (nausea, vomiting, stomatitis), elevated liver enzymes, haematologic abnormalities (e.g. leukopenia) and rare pulmonary or infectious complications. In this study, all anticipated and unexpected adverse events (AEs) are recorded throughout participation. Expected harms include those commonly reported with MTX or GC therapy, while unexpected harms are any other events not described in product information or standard care. AEs are assessed systematically at each study visit and coded using standardised terminology (MedDRA). All AEs are captured in the study database, and summaries of clinically relevant harms will be reported in trial publications.

### Participant timeline {18}

**Fig. 1:**
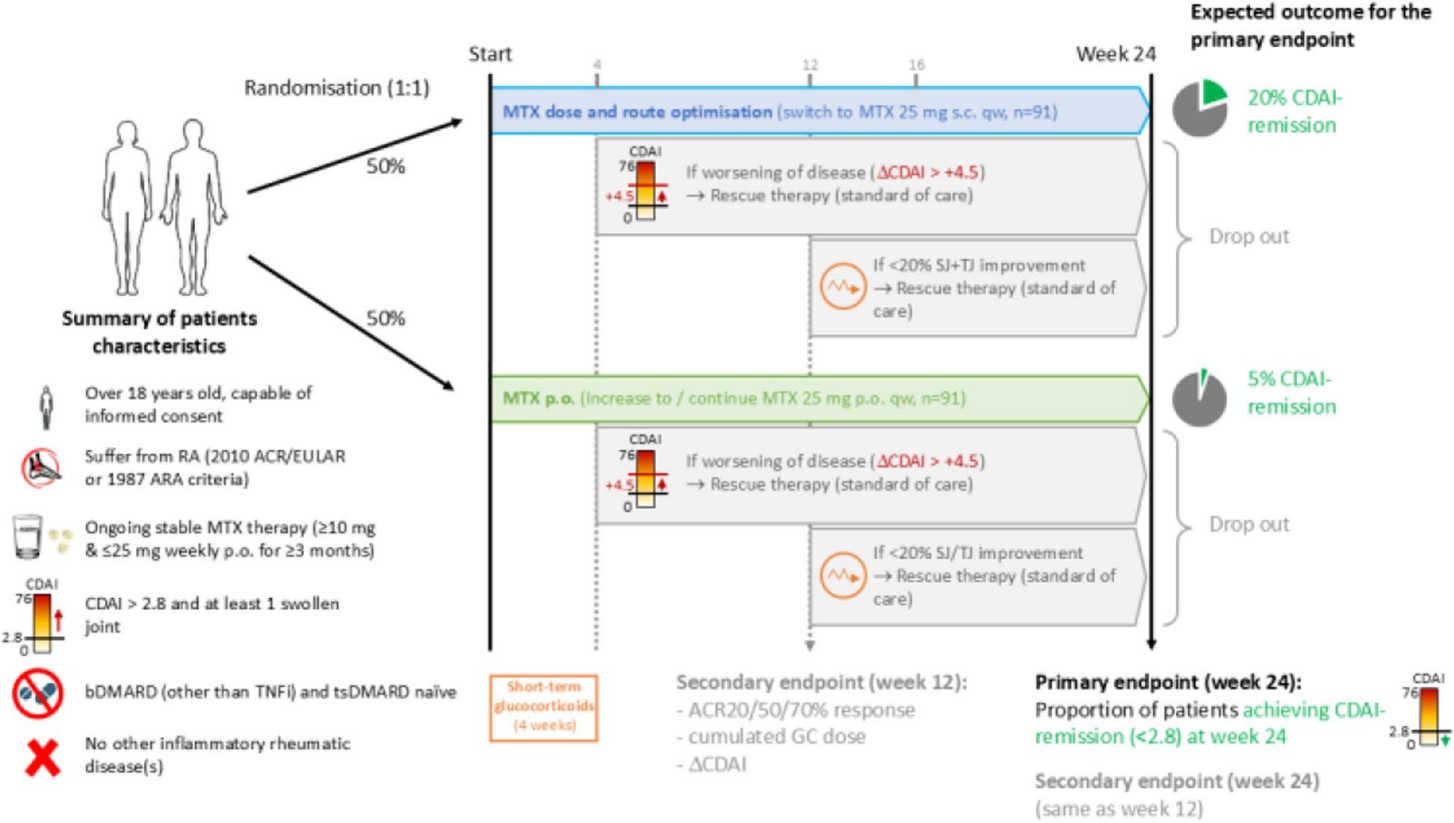
Study flow chart, providing overview of the main inclusion/exclusion criteria, study assignment, efficacy assessments and main outcomes

The study flow chart is outlined in Fig. 1 and the study related assessments in Table 2 (SPIRIT figure). After the screening and baseline visits, patients undergo scheduled study visits at weeks 4, 12, 16 and 24 (± 7 days). At each visit, a blinded joint assessment, patient reported outcomes as well as inflammatory and safety laboratory parameters are performed. Patients are asked to complete three questionnaires: Health Assessment Questionnaire Disability Index (HAQ-DI), the 36-Item Short Form Survey (SF-36) and Functional Assessment of Chronic Illness Therapy (FACIT-Fatigue) to assess the patient-reported outcomes of disease activity and quality of life three times during the study period. At predefined timepoints, drug monitoring is conducted using measurements of MTX-metabolites and TTV in blood. The medication adherence is assessed via an electronic and paper-based adherence assessments and exploratory laboratory measurements. In total, patients undergoing all scheduled study visits remain in the trial for 24 weeks, when primary endpoint is assessed.

**Table 2:** Visit and assessment schedule (SPIRIT figure) according to the protocol version 2.0. Detailed description of laboratory parameters is found in section Data collection

Allowed visit window ± 7 days	**Screening (week-2)**	Baseline visit week 0	Visit week 4	**Visit week 12**	Visit week 16	**Visit week 24**
Written Informed Consent	**x**					
Demography and medical history	**x**					
Record RA classification criteria	**x**					
Inclusion/Exclusion Criteria	**x**	**x**				
Randomisation		**x**				
Distribution of study drug		**x**	**x**	**x**	**x**	
Concomitant Medications	**x**	**x**	**x**	**x**	**x**	**x**
Optional informed consent for electronic adherence (RheumaBuddy App)	**x**					
**Efficacy Assessments**						
Swollen joint count [31]	**x**	**x**	**x**	**x**	**x**	**x**
Tender joint count [31]	**x**	**x**	**x**	**x**	**x**	**x**
Evaluator global assessment (EGA)	**x**	**x**	**x**	**x**	**x**	**x**
Patient global assessment (PGA)	**x**	**x**	**x**	**x**	**x**	**x**
Patient pain assessment (on NRS)	**x**	**x**	**x**	**x**	**x**	**x**
Morning Stiffness (duration and on NRS)	**x**	**x**	**x**	**x**	**x**	**x**
Fatigue (on NRS)	**x**	**x**	**x**	**x**	**x**	**x**
Efficacy assessment CDAI change*			**x**	**x**	**x**	**x**
Efficacy assessment joint count change 20%*				**x**	**x**	**x**
FACIT-Fatigue Questionnaire		**x**		**x**		**x**
Quality of Life Questionnaire SF-36.v1		**x**		**x**		**x**
Disability Scale Questionnaire HAQ-DI		**x**		**x**		**x**
Adherence questionnaire BAASIS-MTX-visit specific		**x**	**x**	**x**	**x**	**x**
RheumaBuddy App use weekly (consent needed)		**x**	**x**	**x**	**x**	**x**
Patient diary MTX and folic acid weekly		**x**	**x**	**x**	**x**	**x**
Patient diary glucocorticoids daily		**x**	**x**	**(x)**	**(x)**	**(x)**
**Safety Assessments**						
Physical Examination	**x**	**x**				**x**
Symptom oriented physical exam		**x**	**x**	**x**	**x**	**x**
Vital Signs (blood pressure, pulse, temperature)	**x**	**x**		**x**		**x**
Symptom-directed exam	**x**	**x**	**x**	**x**	**x**	**x**
Adverse Events		**x**	**x**	**x**	**x**	**x**
**Laboratory Assessments**						
Haematology^1^	**x**^^^	**x**	**x**	**x**	**x**	**x**
Serum Chemistry^2^	**x**^^^	**x**	**x**	**x**	**x**	**x**
CRP & ESR	**x**^^^	**x**	**x**	**x**	**x**	**x**
Pregnancy Test^3^	**x**	**x**	**x**	**x**	**x**	**x**
Hepatitis B and C testing^4^	**x**					
RF and anti-CCP^4^	**x**					
MTX-Polyglutamate sampling		**x**	**x**	**x**		**x**
Torque teno virus sampling		**x**	**x**	**x**		**x**
Biobank and PBMC collection optional		**x**	**x**	**x**		**x**
Sweat analysis sampling incl. diary		**x**		**x**		**x**

^*^In case of a disease flare, patient will be transferred to standard of care and an optional safety follow-up visit after 4 weeks will be scheduled

^1^Red blood cell count (RBC), haemoglobin (Hb), haematocrit (Hct), white blood cell count (WBC), platelet count (PLT)

^2^Creatinine (Crea), blood urea nitrogen (BUN), estimated glomerular filtration rate (eGFR), alanine aminotransferase (AST), aspartate aminotransferase (ALT), gamma-glutamyl transferase (GGT), alkaline phosphatase (AP), total bilirubin, albumin

^^^Window for blood assessment −7 days to 0

^3^Blood or urine; in women of childbearing potential at each study visit at the centre and self-reported test in weeks 8 and 20

^4^Within 6 months prior to screening

### Sample size {19}

Based on literature, the primary endpoint (CDAI ≤ 2.8, i.e. remission) is expected to be achieved by 5% of patients included in the p.o. dose arm, and by 20% in the s.c. arm of the trial [14, 32, 33]. Patients will be randomised in a 1:1 ratio, resulting in an expected sample size for the main outcome of 76 per group (based on *χ*^2^ statistics) with 80% power and a two-sided alpha of 0.05. To account for a conservative 16% dropout rate, the study aims to include 182 patients in total.

### Recruitment {20}

Potential participants with a documented diagnosis of RA who attend routine medical appointments at the co-sponsor sites are pre-screened by study personnel and approached for participation during a regular clinic visit. Potential participants may also be identified through referrals to the study centres. In Vienna, the referral platform Preferrix is used.

## Assignment of interventions: randomisation

### Sequence generation: who will generate the sequence {21a}

The random allocation sequence is generated by unblinded personnel using RANDOMIZER software (Institute for Medical Informatics, Statistics and Documentation, Medical University of Graz, Austria).

### Sequence generation: type of randomisation {21b}

Patients are randomised in a 1:1 ratio, using stratified randomisation by study centre. Details of the software’s randomisation algorithm and any blocking to reduce predictability are documented internally and are not accessible to personnel enrolling participants or assigning interventions.

### Allocation concealment mechanism {22}

Allocation is implemented centrally via the RANDOMIZER software with personalised user accounts for unblinded personnel. The sequence is concealed from joint assessors, who remain blinded to the study assignment throughout the trial.

### Implementation {23}

Personnel enrolling participants do not have access to the random allocation sequence. Randomisation is performed by unblinded personnel with personalised accounts, while the joint assessors who perform outcome measurements remain blinded.

## Assignment of interventions: blinding

### Who will be blinded {24a}

Joint assessors performing outcome measurements are blinded to participants’ study assignments. Patients, care providers and personnel performing randomisation are not blinded.

### How will blinding be achieved {24b}

Blinding is maintained by ensuring that joint assessors do not have access to the random allocation sequence in the RANDOMIZER software and do not communicate with unblinded study personnel or participants about any study-related details. Joint assessors also do not have access to patient data or electronic case report forms.

### Procedure for unblinding if needed {24c}

No emergency unblinding procedures are required, as the trial design does not involve blinded treatment administration. In the unlikely event that unblinding is necessary for safety or clinical reasons, it will be performed by the unblinded study personnel responsible for randomisation and documented in the study records.

## Data collection and management

### Plans for assessment and collection of outcomes {25a}

The following data is collected: demography (age, sex, height, weight, body mass index, ethnic origin, and childbearing potential), medical history (RA, relevant comorbidities), medication history (MTX, folic acid, GC, NSAID and other concomitant medication), disease activity of RA (pain, stiffness, patient global assessment, evaluator global assessment, tender joints, swollen joints, CDAI) and physical examination. The quality of life and subjective disease assessments are performed using questionnaires: HAQ-DI, SF-36 and FACIT-Fatigue. The treatment adherence is assessed with the Basel Assessment of Adherence to Immunosuppressive Medication Scale (BAASIS)-MTX questionnaire, entries within the mobile application RheumaBuddy and the measurement of biomarkers. Haematologic laboratory parameters include red blood cell count (RBC), haemoglobin (Hb), haematocrit (Hct), white blood cell count (WBC), platelet count (PLT). The serum chemistry includes creatinine (Crea), blood urea nitrogen (BUN), estimated glomerular filtration rate (eGFR), creatine kinase (CK), lactate dehydrogenase (LDH), alanine aminotransferase (ALT), aspartate aminotransferase (AST), gamma-glutamyl transferase (GGT), alkaline phosphatase (AP), total bilirubin and albumin. Tests for hepatitis B and C, as well as the rheumatoid factor and anti-CCP antibodies, are performed at screening, unless there is an available result less than 6 months old. Pregnancy test is performed in women of childbearing potential at each study visit at the centre as either urinary or blood test, except for a urinary test at home at weeks 8 and 20, which is self-reported in a diary. Vital signs are assessed regularly.

Exploratory laboratory assessments include the plasma metabolites MTX and 7-hydroxymethotrexate (7-OHMTX), the intracellular MTX-PGs, TTV in plasma, as well as finger sweat.

### Plans to promote participant retention and complete follow-up {25b}

To promote participant retention and complete follow-up, the study visits are scheduled to coincide with routine clinical appointments whenever possible, with additional visits for necessary measurements of clinical activity and biomarkers. Study staff follow up on missed appointments promptly. Efforts are made to maintain regular communication with participants to encourage continued participation and ensure data completeness.

### Data management {26}

All data are transferred from source documents into the electronic Case Report Form (eCRF) Clincase. Data quality is ensured through range and consistency checks, and key variables are verified by independent monitoring.

### Confidentiality {33}

Participant data are pseudonymised, with access restricted to authorised personnel, and securely stored in compliance with data protection regulations.

## Statistical methods

### Statistical methods for primary and secondary outcomes {27a}

The null hypothesis is as follows: The use of 25 mg SCMTX is not superior to 25 mg POMTX weekly in patients with active RA, previously treated with POMTX.

The primary endpoint, defined as the proportion of patients achieving CDAI remission at week 24, will be compared between treatment groups using a *χ*^2^ test. A Fisher’s exact test will be used if the endpoint occurs in fewer than five patients in any group. Superiority of the subcutaneous MTX arm will be tested at a two-sided alpha level of 0.05.

Secondary categorical outcomes will be analysed using *χ*^2^ tests (or Fisher’s exact tests where appropriate), and non-normally distributed continuous outcomes will be analysed using the Mann–Whitney *U* test. For categorical variables, absolute frequencies and percentages will be reported. Time to CDAI remission will be analysed using Kaplan–Meier curves, reporting medians and interquartile ranges, and compared between treatment groups using a log-rank test.

Secondary endpoints will be tested in a pre-specified stepwise hierarchical order until the first non-significant result is reached. All outcomes tested thereafter will be considered exploratory (hypothesis-generating). The following sequence for secondary endpoint testing will be utilised: Proportion of CDAI remission at week 12 > CDAI low disease activity (≤ 10) at week 24 > CDAI low disease activity (≤ 10) at week 12 > ACR70% response at week 24 > ACR50% response at week 24 > Difference in change of patient global assessment > HAQ-DI > CRP at week 24 respectively. An early analysis of the main secondary endpoints will be performed at week 12. All other secondary outcomes will be analysed exploratorily.

### Who will be included in each analysis {27b}

Two analysis sets are defined. The (modified) intention-to-treat (ITT) population includes all randomised participants who received at least one dose of study drug and will be used for all primary analyses. Participants will be analysed according to their allocated treatment group. Non-responder imputation will be applied for patients who discontinue the study or are lost to follow-up over the 24-week study period. The per-protocol population comprises participants who received at least one dose of study drug and did not have major protocol violations that could affect evaluation of the treatment effect on the primary objective. Analyses in this population will be performed as secondary analyses. The time-to-event analysis of time to CDAI remission will not follow a strict ITT approach, as the Kaplan–Meier method accounts for participants who discontinue or are lost to follow-up through censoring.

### How missing data will be handled in the analysis {27c}

For the primary endpoint, non-responder imputation will be applied to all participants who discontinue the study or are lost to follow-up. In time-to-event analyses, missing data due to dropout will be handled by censoring. No additional imputation methods are planned.

### Methods for additional analyses (e.g. subgroup analyses) {27d}

A per-protocol analysis will be conducted as a secondary analysis. No formal subgroup analyses are planned. All additional analyses beyond the pre-specified hierarchical testing strategy will be considered exploratory.

### Interim analyses {28b}

No formal interim analyses or stopping guidelines are planned. The early analysis conducted at week 12 is limited to selected secondary endpoints and will not influence decisions regarding trial continuation or termination.

### Protocol and statistical analysis plan {5}

The full trial protocol, including the pre-specified statistical analysis plan, is published with this manuscript.

## Oversight and monitoring

### Composition of the coordinating centre and Trial Steering Committee {3d}

The trial is coordinated by the sponsor, Medical University of Vienna, which is responsible for overall trial management, regulatory compliance, communication with participating sites and day-to-day operational support. The coordinating team meets with co-sponsors bi-weekly to review recruitment, data quality, and operational issues.

A Trial Steering Committee (TSC) provides overall scientific oversight of the trial. The TSC is responsible for monitoring trial progress, adherence to the protocol, and safeguarding the scientific integrity of the study. The committee meets at least once monthly over the course of the trial and provides guidance to the participating centres as required. No independent endpoint adjudication committee is planned, as outcome assessment is performed by blinded joint assessors according to predefined criteria.

### Composition of the data monitoring committee, its role and reporting structure {28a}

Given the low-risk nature of the intervention, a formal independent Data and Safety Monitoring Board (DSMB) has not been established. Safety oversight is ensured by the principal investigator and study personnel, with systematic recording and assessment of adverse events, timely reporting of serious adverse events, and external oversight via local monitors, CTIS, and potential regulatory or ethics committee inspections.

### Frequency and plans for auditing trial conduct {29}

Trial conduct and data quality are monitored on a regular basis by the respective co-sponsor. Monitoring activities include review of data completeness and consistency, verification of key study variables, and assessment of protocol compliance. Audits by institutional or regulatory authorities may be conducted at any time. No additional independent auditing procedures are planned.

### Protocol amendments {31}

To address slow patient enrolment, the potential patient pool was expanded by changing the exclusion criterion number 2 from “Ongoing or previous therapy with any biological DiseaseModifying Anti-Rheumatic Drug (bDMARDs) or targeted synthetic DMARDs (tsDMARDs) or conventional synthetic DMARDs (csDMARDs) other than methotrexate and Hydroxychloroquine” to “Ongoing or previous therapy with any targeted synthetic DMARDs (tsDMARD) or biological Disease-Modifying Anti-Rheumatic Drug (bDMARD) with the exception of any TNFα inhibitor in a stable dose and interval for at least 4 months; We allow ongoing or previous therapy with a conventional synthetic DMARD (csDMARD) with a stable dose in the past 4 months”. Also, the visit week 8 was removed from the schedule to minimise the burden of frequent visits for patients. The future study timelines were adapted according to the regulatory delay and recruitment rates. Albumin was added to the chemistry parameters as a relevant factor for MTX metabolite analysis. Two new cosponsor centres were added: Milan, Italy, and Bucharest, Romania.

Any further protocol amendments will be submitted for review and approval by the relevant authorities listed above and information updated on ClinicalTrials.gov.

### Dissemination policy {8}

Trial results will be disseminated through peer-reviewed publications, scientific presentations and reporting in the trial registry, with study findings made available to participants on request.

## Discussion

Despite the fact that MTX is proven to be safe, efficient and cost-effective, it is likely that it does not achieve its full potential in clinical practice. The results of this trial will provide further insights into the clinical efficacy of the subcutaneous versus oral MTX when increased to 25 mg weekly in patients with active rheumatoid arthritis, who are naïve to b/tsDMARDs. Our aim is to generate data that support clinical decision-making in optimisation of MTX dosing and administration routes for patients not being on target, and to establish evidence on novel biomarkers and their association with efficacy, hopefully paving the way for personalised MTX treatment strategies.

The MethMax trial is an investigator-initiated trial, part of Horizon Europe and funded by the Squeeze project. The Squeeze project initiative aims to optimise the use of existing therapies for rheumatic diseases through three distinct clinical trials, including MethMax. By including seven different European countries (Austria, Sweden, Norway, Netherlands, Italy, Romania and the UK), this trial covers diverse geographical patient pools, health care settings, and socioeconomic aspects of MTX prescription.

There are multiple reasons, which might limit the efficacy of oral MTX therapy: insufficient dosing, inadequate treatment adherence and subsequent potentially premature switch to b/tsDMARDs. In MethMax, we aim to address all these issues. Factors affecting patients’ treatment adherence may include side effects, the complexity of drug regimens or inadequate communication between physician and patient regarding the disease progression and treatment plan [34]. To truly compare the therapy effects of oral and subcutaneous arm based solely on their pharmacological effects and not on differences in treatment adherence that could exist, we will monitor thoroughly, and potentially even raise treatment adherence by using patient medication diaries, adherence questionnaires, digital medication tracking as well as objective methods like biomarkers. None of the existing standard methods to measure DMARD adherence is free from limitations; therefore, a combination of these three complementary measures will increase validity and reliability. To achieve this, the BAASIS questionnaire [35], originally validated in cohorts of organ-transplanted patients and used in multiple trials assessing adherence [36], was adapted to MethMax. This trial will be the first study to investigate the BAASIS-MTX, an adapted version to specifically assess MTX adherence. Importantly, we expect that the sum of adherence measurement methods used in this trial will increase the interpretation of clinical outcomes.

MTX-PGs have been investigated as active intracellular metabolites which could be utilised as a predictor of clinical outcomes and individual treatment response characteristics. Given the low concentrations of these metabolites in red blood cells and their limited stability, test methods allowing further use paired with thorough pre-analytics and logistics are necessary in clinical trials. Ongoing research is currently investigating TDM with individual MTX dosing based on MTX-PG_3–5_ levels and factors identifying MTX exposure–response association [37]. One recent study in particular in MTX-naïve patients started on MTX showed that higher MTX-PG concentrations were an independent factor for lower disease activity in MTX monotherapy, but not after adalimumab initiation in patients with continued MTX dose [38]. Thus, more data is needed to explore the behaviour of MTX metabolites with different therapy regimens.

Overall, the strength of the MethMax trial lies in its study design and procedures, combining multiple objective and subjective measurements of disease activity, patient outcomes and treatment adherence. The efforts to maximise the therapy adherence should deliver more representative results on the true pharmacological effects of MTX in its distinct routes. The main limitations of this study could include recruitment problems due to the relatively stringent inclusion/exclusion criteria, reliance on self-reported and self-performed data, and the impact of daily variations of subjective disease activity, especially in patients with an initial low disease activity score. Furthermore, patients and physicians are unblinded to the treatment allocation, and only joint assessment is performed by blinded personnel.

The anticipated results might bring valuable knowledge about the “maximisation” of the MTX therapy potential in patients with RA, providing recommendations on the optimal dose and administration route.

## Trial status

The first approved protocol version 1.1 was from 28th March 2024. The latest approved version is 2.0 from 16th May, which was approved on 11th August 2025. The first patient was enrolled at the Medical University of Vienna on 12th August 2024. The first visit of the last patient is planned for the end of November 2026. The last patient’s visit is therefore expected to occur approximately 24 weeks later, in May 2027.

## Data Availability

Principal investigators, sponsors and statisticians will have access to the final dataset. More detailed information can be provided upon reasonable request.

## Abbreviations

ACR: American College of Rheumatology
AICAR: 5-Aminoimidazole-4-carboxamide ribonucleotide
ALT: Alanine aminotransferase
AST: Aspartate aminotransferase
ATIC: 5-Aminoimidazole-4-carboxamide ribonucleotide transformylase / inosine monophosphate cyclohydrolase
bDMARD: Biological disease-modifying antirheumatic drug
CDAI: Clinical Disease Activity Index
CDAI-REM: Clinical Disease Activity Index remission
CRP: C-reactive protein
csDMARD: Conventional synthetic disease-modifying antirheumatic drug
CTIS: Clinical Trials Information System
DMARD: Disease-modifying antirheumatic drug
DSMB: Data and Safety Monitoring Board
ECRF: Electronic Case Report Form
EMA: European Medicines Agency
ESR: Erythrocyte sedimentation rate
EOS: End of study
EULAR: European Alliance of Associations for Rheumatology
FACIT-F: The Functional Assessment of Chronic Illness Therapy – Fatigue
GC: Glucocorticoids
GCP: Good clinical practice
HAQ-DI: The Health Assessment Questionnaire-Disability Index
ICH-GCP: International Council for Harmonisation of Technical Requirements for Pharmaceuticals for Human Use—Good Clinical Practice
ITT: Intention to treat
KKS: Koordinationszentrum für Klinische Studien
MTX: Methotrexate
MTX-PG: Methotrexate polyglutamate
NSAID: Non-steroidal anti-inflammatory drug
PBMC: Peripheral blood mononuclear cell
PGA: Patient Global Assessment
PLT: Platelets
p.o.: Per os/per oral
QMUL: Queen Mary University of London
RA: Rheumatoid arthritis
RBC: Red blood cells
s.c.: Subcutaneous
SF-36v1: 36-Item Short Form Survey version 1
TDM: Therapeutic drug monitoring
TNFi: Tumor necrosis factor inhibitor
TSC: Trial Steering Committee
tsDMARD: Targeted synthetic disease-modifying antirheumatic drug
TTV: Torque teno virus
WBC: White blood cells
